# MRI-derived habitat heterogeneity for overall survival risk stratification in IDH-wildtype, CNS WHO grade 4 glioblastoma

**DOI:** 10.3389/fcell.2026.1917791

**Published:** 2026-09-09

**Authors:** Xuhao Dai, Luyan Sun, Ting Zhu, Jiazhen Zhu, Yuqing Hu, Xiaoqin Ge, Ruishuang Ma, Shengping Gong, Jiming Yang, Yingying Zhou, Hongwei Li, Yipeng Song, Qingsong Tao, Jiangping Ren

**Affiliations:** 1 Department of Radiotherapy and Chemotherapy, The First Affiliated Hospital of Ningbo University, Ningbo, China; 2 Department of Radiotherapy, Yantai Yuhuangding Hospital, Yantai, China; 3 Department of Radiology, Ningbo No. 2 Hospital, Ningbo, China; 4 Central Laboratory, The First Affiliated Hospital of Ningbo University, Ningbo, China

**Keywords:** IDH-wildtype glioblastoma, MRI habitat analysis, overall survival, prognostic modeling, radiomics, spatial heterogeneity

## Abstract

**Background:**

Glioblastoma (GBM), IDH-wildtype, CNS WHO grade 4 has marked spatial heterogeneity, yet routine MRI-based prognostic assessment often relies on whole-tumor summaries. We developed a preoperative MRI habitat-analysis framework to quantify intratumoral and peritumoral spatial phenotypes and to evaluate their value for overall survival (OS).

**Methods:**

This retrospective multicohort study included a development cohort of 473 patients assembled from the UCSF-PDGM-v5 dataset (n = 354) and Yantai Yuhuangding Hospital (n = 119), and an independent external testing cohort from the First Affiliated Hospital of Ningbo University (n = 82). All habitat generation, selection of the optimal habitat number, feature selection, and model development were performed using the pooled development cohort. Multiparametric preoperative MRI was partitioned into voxel-wise habitats using k-means clustering. Habitat-derived variables were selected and combined into a risk score using LASSO-Cox modeling. Baseline, habitat, and combined prognostic models were constructed using Cox proportional hazards regression and evaluated using the C-index, time-dependent AUC, calibration, prediction-error analysis, decision curve analysis, and Kaplan–Meier risk stratification.

**Results:**

A four-habitat solution was stable and biologically interpretable. Necrotic-like and edema-dominant peripheral habitats showed the strongest adverse associations with OS. The habitat risk score remained independently prognostic after adjustment for the consistently available baseline variables of age, sex, and MGMT promoter methylation. In external testing, the combined model improved the external C-index from 0.604 to 0.701 and the 12-month AUC from 0.626 to 0.724, with reduced prediction error, improved calibration, and separated high- and low-risk groups.

**Conclusion:**

MRI-derived habitat analysis captures survival-relevant spatial heterogeneity in GBM and provides an interpretable, noninvasive approach for risk stratification warranting prospective validation.

## Introduction

Glioblastoma (GBM), IDH-wildtype, CNS WHO grade 4 remains the most aggressive adult diffuse glioma despite advances in molecular classification and multimodality treatment. The 2021 WHO framework has clarified the diagnostic distinction between IDH-mutant astrocytoma and GBM, yet substantial survival heterogeneity persists among patients carrying the same integrated diagnosis (Louis et al., 2021; Weller et al., 2021; Wen et al., 2023; Ellingson et al., 2024; Wen and Packer, 2021; Antonelli and Poliani, 2022). This clinical variation suggests that prognosis is shaped not only by histomolecular taxonomy and conventional baseline variables, but also by the spatial organization of tumor and peritumoral microenvironments that are only partially captured by routine radiologic descriptors.

GBM is increasingly understood as an ecosystem composed of malignant cell states, reactive glial populations, vascular and hypoxic niches, immune infiltrates, and treatment-resistant compartments (Ruiz-Moreno et al., 2025; Greenwald et al., 2024; Ravi et al., 2022; Coy et al., 2022; Mathur et al., 2024; Lv et al., 2024). Tissue-based spatial and single-cell approaches can resolve these biological architectures, nevertheless, they are not suitable for repeated whole-lesion assessment in routine practice. Multiparametric MRI provides a noninvasive view of the entire tumor-bearing region and has been linked to molecular, immune, and prognostic phenotypes (Fathi Kazerooni et al., 2025; Verma et al., 2022; Styliara et al., 2024; Hao et al., 2025). However, many radiomic pipelines still aggregate features across the whole-tumor volume. Such global summaries may dilute clinically important subregional information from enhancing tumor, necrotic core, edema-dominant periphery, and relatively preserved solid components.

MRI habitat imaging addresses this limitation by partitioning multiparametric scans into subregions with shared signal characteristics. Instead of treating intratumoral variation as noise, habitat analysis explicitly quantifies the composition and spatial arrangement of imaging-defined niches (Yang et al., 2022; Waqar et al., 2022; Wang, 2025; Yang et al., 2025; Han et al., 2026; Niu et al., 2025; Folty et al., 2025; Bentourkia and Abdo, 2025). Prior glioma studies have suggested that necrotic, edema-associated, and texture-defined subregions may relate to molecular risk, recurrence patterns, and survival. Nevertheless, externally tested habitat frameworks for GBM remain limited, and few studies have evaluated habitat reproducibility, interpretability, independent prognostic value, incremental performance over baseline variables, and risk stratification within a single multicohort design.

We therefore developed a preoperative MRI habitat-analysis framework for GBM using a multicenter development cohort and an independent external testing cohort. We aimed to determine whether MRI-defined habitats identify reproducible and survival-associated spatial phenotypes, whether a habitat-derived risk score provides information beyond available clinical and molecular variables, and whether habitat-informed models improve noninvasive OS prediction and clinically interpretable risk stratification.

This focus on spatial organization is particularly relevant for GBM in that distinct radiologic compartments may reflect disparate underlying biological processes. Enhancing solid tumor may correspond to blood–brain barrier disruption and cellular tumor burden, low-enhancement central regions may reflect necrosis and hypoxia, and FLAIR-hyperintense peritumoral regions may contain edema, infiltrating tumor cells, vascular changes, and treatment-resistant niches. A clinically useful habitat model should therefore transcend mere statistical improvement. Specifically, it ought to generate subregions that demonstrate cross-cohort stability, yield interpretable imaging correlates, and deliver incremental prognostic value in independent external populations.

## Methods

### Study design and patient cohorts

This retrospective multicohort study evaluated whether preoperative MRI-defined habitats capture prognostically relevant spatial heterogeneity in GBM. The development cohort comprised 473 patients from the UCSF-PDGM-v5 dataset available through The Cancer Imaging Archive (n = 354) and Yantai Yuhuangding Hospital (n = 119). The independent external testing cohort comprised 82 patients from the First Affiliated Hospital of Ningbo University. The UCSF-PDGM-v5 dataset was used in accordance with TCIA data-use requirements (Calabrese et al., 2022). The two development-cohort sources were pooled for habitat generation, selection of the optimal habitat number, feature selection, and model construction. The external testing cohort was reserved *a priori* and was not used for habitat selection, parameter estimation, or model optimization.

Eligible patients were required to have a pathologically confirmed diagnosis of GBM, IDH-wildtype status, preoperative MRI suitable for habitat analysis, and available OS time and event-status information. Patients with IDH-mutant or unknown IDH status were excluded from the primary analysis, as were cases with inadequate imaging or incomplete survival information. The final development cohort included 473 patients, and the external testing cohort included 82 patients. Baseline variables consistently available across cohorts included age, sex, and MGMT promoter methylation status. Because extent-of-resection data were unavailable in the external testing cohort, this variable was not included in the baseline model, although descriptive information was reported where available.

The modeling strategy was designed to preserve a strict separation between development and external testing. Variables were restricted to information derived from preoperative MRI or consistently available across cohorts. The external testing cohort was not used to define the habitats, select the number of habitats, screen features, estimate model coefficients, or optimize model performance. This separation was intended to approximate application of a model developed from reference data to an independent institution with different acquisition conditions and patient characteristics.

### MRI segmentation and preprocessing

Preoperative contrast-enhanced T1-weighted imaging, T2-weighted imaging, and FLAIR imaging were used for tumor delineation and habitat construction. The tumor-bearing region was defined as the whole-tumor region, including the enhancing tumor, non-enhancing/necrotic tumor core, and peritumoral T2/FLAIR-hyperintense region. For the UCSF-PDGM-v5 cohort, the publicly available tumor masks were used after visual quality assessment and manual correction when necessary. For the institutional cohorts, tumor segmentation was performed using the Segment Editor module in 3D Slicer (version 5.6.2) through semi-automatic delineation followed by slice-by-slice manual refinement. The contours were independently reviewed by two board-certified radiologists with 8 and 12 years of neuroimaging experience, respectively. Disagreements regarding tumor boundaries were resolved by consensus.

Before habitat analysis, N4 bias-field correction was performed using Advanced Normalization Tools (ANTs, version 2.5.0), and skull stripping was performed using HD-BET (version 1.0). For each patient, the contrast-enhanced T1-weighted image was used as the reference space. T2-weighted and FLAIR images were registered to the contrast-enhanced T1-weighted image using six-degree-of-freedom rigid registration with mutual information as the similarity metric. All MRI sequences were subsequently resampled to an isotropic voxel spacing of 1 × 1 × 1 mm^3^. Linear interpolation was used for MRI images, whereas nearest-neighbor interpolation was used for segmentation masks to preserve discrete label values. Within the tumor-bearing region, voxel intensities from each MRI sequence were standardized separately using z-score normalization. Registration accuracy, skull-stripping results, image quality, and tumor-mask alignment were visually inspected for all patients. Cases with severe motion artifacts, incomplete sequence coverage, failed registration, or irreparable segmentation errors were excluded, while correctable cases were reprocessed before habitat analysis.

### Habitat generation and feature extraction

Habitat generation and feature extraction were performed in Python 3.10.13 using NumPy 1.26.4, SciPy 1.11.4, scikit-learn 1.3.2, scikit-image 0.22.0, and SimpleITK 2.3.1. For each patient, voxel intensities from contrast-enhanced T1-weighted, T2-weighted, and FLAIR images within the tumor-bearing region were standardized separately using z-score normalization. To prevent patients with larger tumors from disproportionately influencing clustering, an equal number of voxels was randomly sampled from each patient in the development cohort.

The sampled voxel-level multiparametric intensity vectors from the pooled development cohort were clustered using k-means++ initialization with Euclidean distance, 50 initializations, a maximum of 500 iterations, and a fixed random seed of 42. Candidate habitat numbers from k = 2 to five were evaluated. A single cluster (k = 1) was excluded because it cannot represent intratumoral spatial heterogeneity, whereas an upper limit of five habitats was selected to balance biological granularity, reproducibility, and interpretability while limiting overpartitioning into small and potentially unstable compartments. Candidate solutions were compared using the Silhouette score, Calinski–Harabasz index, Davies–Bouldin index, bootstrap stability based on 1,000 resampling iterations, survival association, and anatomical interpretability. The optimal habitat number was selected entirely within the development cohort. The resulting cluster centroids were then fixed and used to assign voxels in the external testing cohort to the nearest habitat without recalibration.

Habitat-derived features were extracted from the resulting three-dimensional habitat maps. These included the fractional volume of each habitat, habitat entropy, spatial dispersion, necrotic-to-solid habitat ratio, enhancing-to-edema habitat ratio, habitat volume, distance to the tumor centroid, boundary complexity, necrosis overlap, and edema overlap. Habitat fractions were calculated as the number of voxels belonging to each habitat divided by the total number of voxels within the tumor-bearing region. Habitat entropy was calculated as $-\sum p_{i}\log p_{i}$ , where $p_{i}$ represents the fractional volume of habitat i .

Before feature selection, features with zero variance were removed, highly correlated features were filtered using an absolute Spearman correlation coefficient greater than 0.90, and continuous features were standardized using the mean and standard deviation of the development cohort. Candidate features were screened using univariable Cox regression with Benjamini–Hochberg false-discovery-rate correction. Features with an FDR-adjusted q value below 0.10 were entered into LASSO-Cox regression. The penalty parameter was selected using 10-fold cross-validation and the one-standard-error criterion. The selected features, coefficients, and standardization parameters were fixed before application to the external testing cohort.

### Imaging-based interpretation of MRI habitats

Biological interpretation was performed after completion of unsupervised clustering and did not contribute to cluster generation or selection. For each habitat, the interpretation integrated three complementary sources of information: (1) the cluster-centroid signal profile across contrast-enhanced T1-weighted, T2-weighted, and FLAIR images; (2) the predominant spatial distribution of the habitat within the tumor-bearing region; and (3) its quantitative overlap with conventional necrotic/non-enhancing and peritumoral T2/FLAIR-hyperintense compartments. These imaging patterns were interpreted according to previously reported radiologic–pathologic associations in glioblastoma. Because spatially matched histopathological or molecular sampling was not available, the resulting labels were considered putative imaging-based interpretations rather than histologically confirmed biological identities.

### Risk modeling and model evaluation

Habitat fractions were calculated as the proportion of the tumor region assigned to each habitat. Additional descriptors included habitat entropy, spatial dispersion, necrotic-to-solid habitat ratio, enhancing-to-edema habitat ratio, volume, fractional occupancy, distance to tumor center, boundary complexity, necrosis overlap, and edema overlap. These variables were designed to reflect both the amount of each habitat and the spatial arrangement of aggressive-appearing compartments.

The UCSF-PDGM-v5 and Yantai cohorts were pooled as a single development cohort (n = 473), and the external testing cohort (n = 82) was reserved exclusively for independent evaluation. All habitat generation, selection of k, feature preprocessing, feature selection, coefficient estimation, and model construction were performed using the development cohort only.

Continuous habitat features were standardized using the mean and standard deviation derived from the development cohort. Candidate features were initially screened using univariable Cox regression with Benjamini–Hochberg false-discovery-rate correction and were subsequently entered into LASSO-Cox regression. The penalty parameter was selected using 10-fold cross-validation with the one-standard-error criterion. The selected features, regression coefficients, and standardization parameters were fixed and applied unchanged to the external testing cohort. The final habitat risk score was calculated as:

$$\text{Habitat risk score}=\beta_{1}Z\left({\text{Feature}}_{1}\right)+\beta_{2}Z\left({\text{Feature}}_{2}\right)+\cdots +\beta_{p}Z\left({\text{Feature}}_{p}\right)$$

$$Z_{i}=\left(X_{i}-\mu_{i}\right)/\sigma_{i}$$

Where Feature_i denotes the selected habitat-derived feature, and β_i, μ_i, and σ_i denote the corresponding LASSO-Cox coefficient, development-cohort mean, and development-cohort standard deviation, respectively. The complete equation and corresponding parameters are provided in Supplementary Table S13.

Three time-to-event prognostic models were constructed using Cox proportional hazards regression. The baseline model included age, sex, and MGMT promoter methylation status. The habitat model included the LASSO-Cox-derived habitat risk score, and the combined model incorporated age, sex, MGMT promoter methylation status, and the habitat risk score. Model coefficients were estimated in the development cohort and applied without re-estimation to the external testing cohort. The proportional hazards assumption was assessed for each model. The continuous risk score was retained for discrimination, calibration, prediction-error, and incremental-value analyses. For Kaplan–Meier visualization, patients were categorized into low- and high-risk groups using the median habitat risk score calculated separately within each cohort. Discrimination was quantified using the C-index and time-dependent AUC at 6, 12, 18, and 24 months. Prediction error was assessed using Brier scores and the integrated Brier score. Calibration was summarized using calibration slope, and clinical utility was evaluated using 12-month decision curve analysis.

### Risk stratification and sensitivity analyses

Patients were divided into low- and high-risk groups for Kaplan–Meier visualization using the median habitat-derived risk score within each cohort. These cohort-specific median cutoffs were used only for descriptive risk-group display and log-rank comparisons; the continuous risk score and model coefficients were not re-estimated in the external testing cohort. Univariable and multivariable Cox models estimated HRs, with adjusted models including age, sex, and MGMT promoter methylation status.

Subgroup analyses were restricted to variables available in the analytical data, including age group and MGMT promoter methylation status. Interaction tests assessed whether the habitat effect differed across subgroups. Sensitivity analyses tested alternative habitat numbers, alternative risk cutoffs, public-database-only development followed by external testing, analyses restricted to complete MGMT promoter methylation status, and exclusion of OS outliers. The primary analysis employed the combined model with the optimal habitat number (k = 4). Robustness was judged by consistency of the external C-index, 12-month AUC, high-versus-low risk HR, and log-rank results.

### Statistical analysis

Categorical variables were compared using Fisher’s exact test. OS distributions were compared using log-rank tests. Cox proportional hazards models were used for survival analyses, with HRs reported alongside 95% CIs and p values. The analytical modeling pipeline was specified as follows: k-means clustering was used for voxel-wise habitat generation, LASSO-Cox regression was used for habitat-feature selection and risk-score construction, and Cox proportional hazards regression was used to construct the baseline, habitat, and combined survival models and evaluate survival associations and incremental prognostic value. Model performance was evaluated using the C-index, time-dependent AUC, Brier score, integrated Brier score, calibration slope, and decision curve net benefit. Ninety-five percent confidence intervals for the C-index and time-dependent AUCs were estimated using 1,000 patient-level bootstrap resamples. Incremental value over the baseline model was assessed using changes in C-index and time-dependent AUC, net reclassification improvement, and integrated discrimination improvement. Two-sided p < 0.05 was considered statistically significant unless otherwise specified.

The analytical workflow integrated three hierarchical levels of evidence: (1) assessment of MRI habitat partition reproducibility and interpretability; (2) evaluation of the association between continuous habitat-derived features and overall survival; (3) quantification of the integrated score’s incremental prognostic value over baseline variables for survival modeling. This stepwise strategy was adopted to avoid overreliance on single metrics, and to validate the habitat framework’s robustness across discrimination, calibration, prediction error, clinical utility, and risk-group separation.

## Results

### Cohort construction and baseline characteristics

**FIGURE 1:**
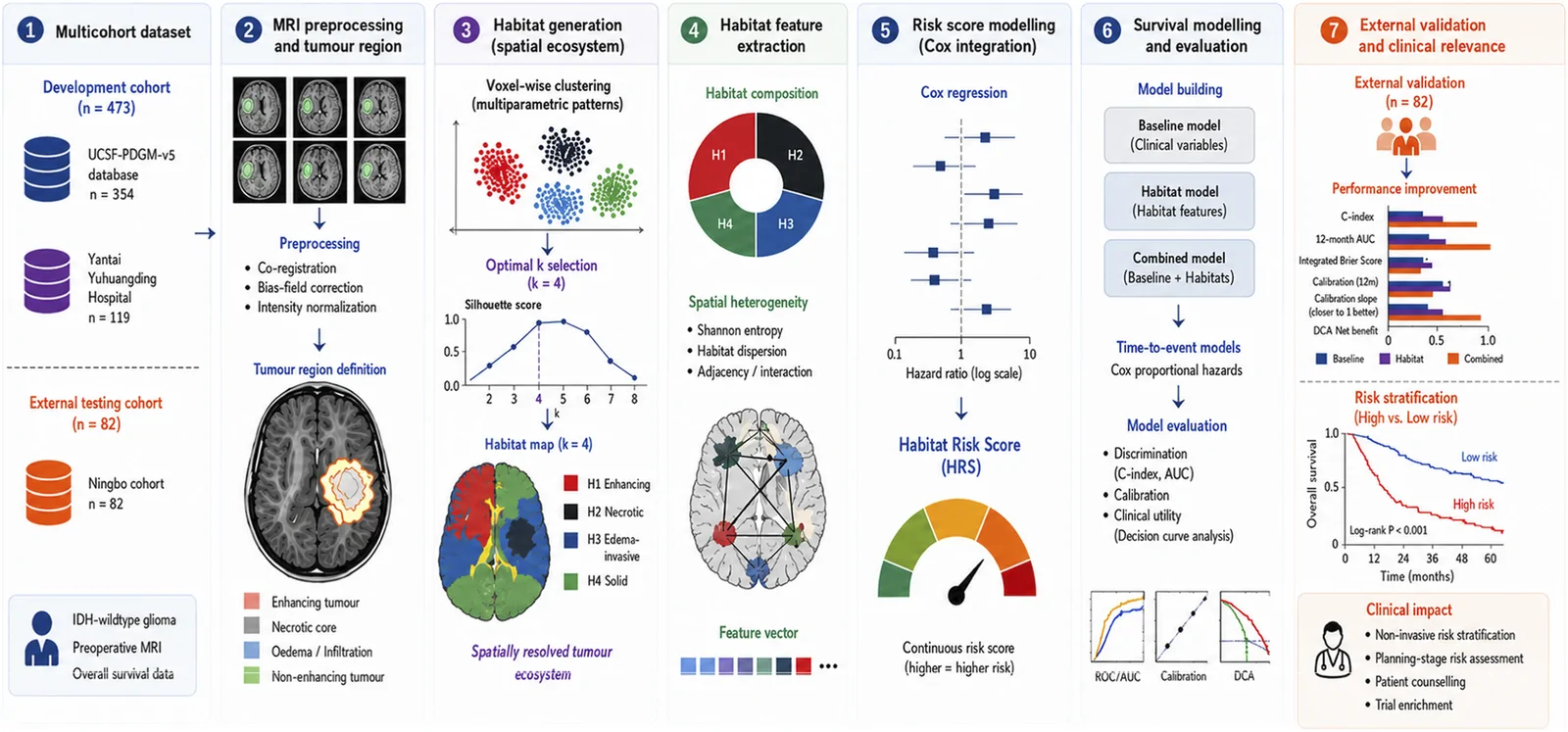
Overall analytical workflow of habitat-based MRI survival modeling. The pooled development cohort was used for MRI preprocessing, voxel-wise habitat generation, selection of k = 4, habitat-feature extraction, LASSO-Cox risk-score construction, and Cox-based prognostic modeling. Performance was evaluated using discrimination, prediction error, calibration, decision curve analysis, and Kaplan-Meier risk stratification, followed by independent external testing without coefficient re-estimation or recalibration.

**FIGURE 2:**
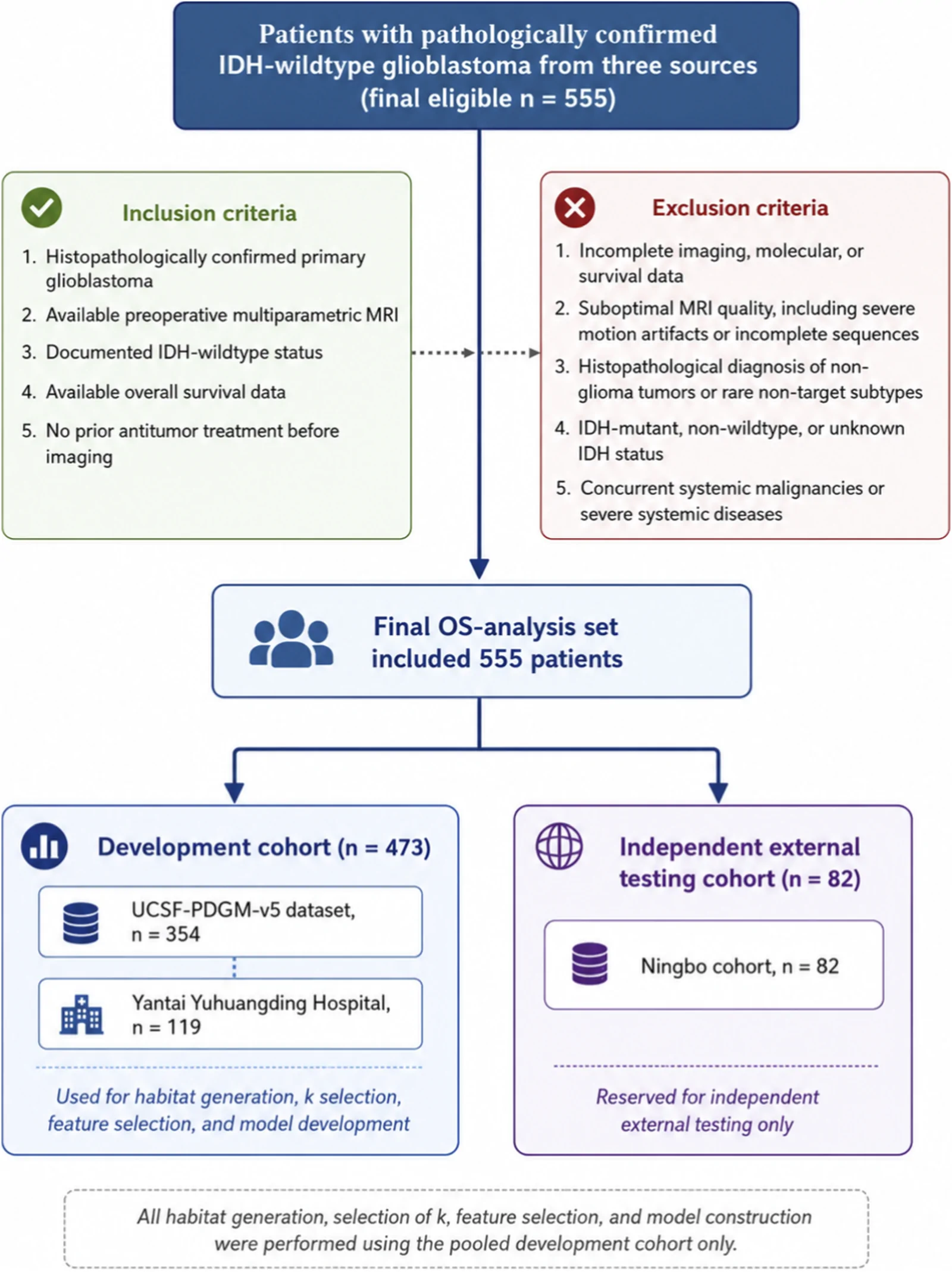
Cohort assembly and eligibility framework. The analytical dataset comprised 555 patients from the UCSF-PDGM-v5 dataset, Yantai Yuhuangding Hospital, and an independent Ningbo cohort. The development cohort included 473 patients (UCSF-PDGM-v5, n = 354; Yantai, n = 119), and the independent external testing cohort included 82 patients.

We first assembled a multicohort dataset to evaluate whether MRI-defined habitats could provide reproducible prognostic information in GBM. Figure 1 shows the analytical workflow, beginning with cohort construction, GBM case selection, MRI habitat generation, survival modeling, and external testing. Figure 2 summarizes cohort assembly and the eligibility framework. The development cohort included 473 patients with GBM and available OS information, comprising 354 patients from UCSF-PDGM-v5 and 119 patients from Yantai Yuhuangding Hospital. The independent external testing cohort included 82 patients (Figure 2; Table 1).

**TABLE 1:** Cohort composition and survival-event distribution.

Cohort	Included n	Glioblastoma, IDH-wildtype, CNS WHO grade 4	IDH-mutant/Excluded for non-IDH-wildtype status	IDH-unknown excluded	OS available n	Death event n (%)	Censored n (%)
Development cohort	473	473	0	0	473	286 (60.5%)	187 (39.5%)
External testing cohort	82	82	0	0	82	67 (81.7%)	15 (18.3%)

In the development cohort, 286 deaths were observed, corresponding to an event rate of 60.5%, compared with 67 deaths and an event rate of 81.7% in the external testing cohort. The external testing cohort therefore had a lower censoring proportion than the development cohort (18.3% vs. 39.5%). Because crude event proportions do not account for the timing of events or censoring, they are not directly equivalent to Kaplan–Meier survival distributions; the cohort-level OS distributions did not differ significantly (log-rank p = 0.058). Median age was 61.0 years in the development cohort and 64.5 years in the external testing cohort, and MGMT promoter methylation frequency was comparable between cohorts. The proportion of female patients was numerically higher in the external testing cohort, although this difference did not reach conventional statistical significance (Table 2).

**TABLE 2:** Baseline characteristics of the development and external testing cohorts.

Variables	Development cohort (n = 473)	External testing cohort (n = 82)	p value
Glioblastoma, IDH-wildtype, CNS WHO grade 4, n (%)	473 (100.0%)	82 (100.0%)	—
Age (years), median (IQR)	61.0 (53.0–69.0)	64.5 (55.0–70.0)	0.641
Sex, n (%)	​	​	0.069
Male	288 (60.9%)	41 (50.0%)	​
Female	185 (39.1%)	41 (50.0%)	​
MGMT promoter methylation status, n (%)	​	​	0.177
Methylated	294 (62.2%)	44 (53.7%)	​
Unmethylated	179 (37.8%)	38 (46.3%)	​
OS event status, n (%)	​	​	<0.001
Death	286 (60.5%)	67 (81.7%)	​
Censored	187 (39.5%)	15 (18.3%)	​

### Habitat generation and spatial interpretation

**FIGURE 3:**
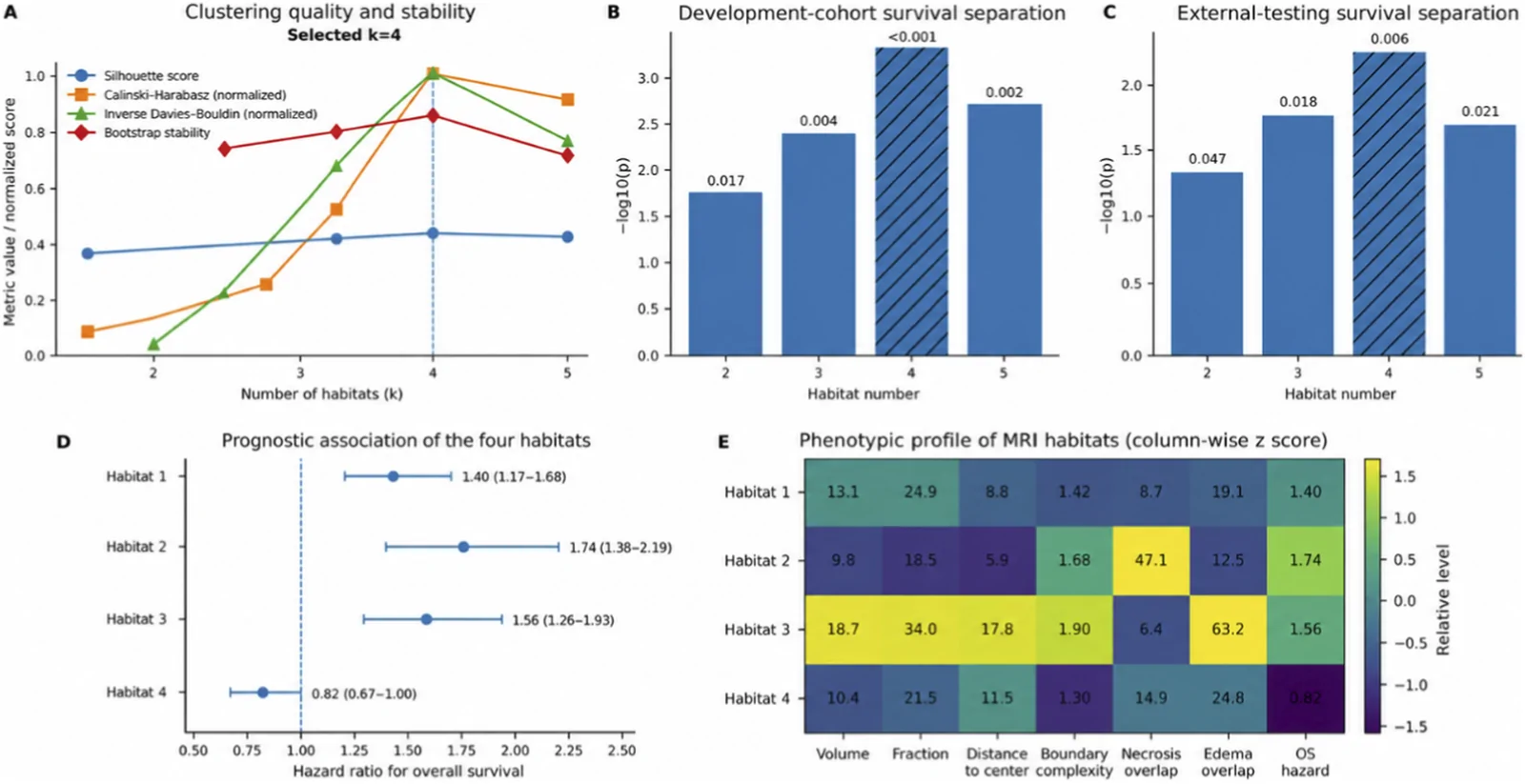
Selection and characterization of MRI-derived tumor habitats **(A)** Clustering quality and stability for candidate k values in the development cohort **(B)** Development-cohort survival separation **(C)** Confirmatory survival separation in the external testing cohort **(D)** Associations of the four habitats with overall survival **(E)** Imaging and spatial phenotype profile of each habitat. HR, hazard ratio; OS, overall survival.

Among candidate habitat numbers evaluated in the development cohort, k = 4 provided the strongest balance of clustering quality, bootstrap stability, survival relevance, and anatomical interpretability. The four-habitat solution had a Silhouette score of 0.448, Calinski–Harabasz index of 1232.5, Davies–Bouldin index of 0.741, and bootstrap stability of 0.85. Its survival relevance was subsequently confirmed in the external testing cohort (Figures 3A–C; Table 3).

**TABLE 3:** Selection of the optimal number of MRI habitats in the development cohort, with confirmatory evaluation in the external testing cohort.

Number of habitats k	Silhouette score	Calinski-Harabasz index	Davies-Bouldin index	Bootstrap stability	Development log-rank p	External log-rank p	Significance	Selected
2	0.386	861.4	0.914	0.78	0.017	0.047	*	No
3	0.429	1054.7	0.792	0.82	0.004	0.018	**	No
4	0.448	1232.5	0.741	0.85	<0.001	0.006	***	Yes
5	0.435	1196.3	0.773	0.74	0.002	0.021	**	No

The four habitats showed distinct imaging and spatial phenotypes. Habitat 1 showed an enhancing-dominant pattern. Habitat 2 showed a necrotic-like pattern with low enhancement, high T2 signal, and high necrosis overlap. Habitat 3 showed a peripheral edema-dominant pattern with high FLAIR signal, low T1ce signal, and high edema overlap. Habitat 4 showed a mixed solid-tumor pattern with comparatively lower necrotic and edema burden (Figures 3D,E; Table 4). These labels represent putative imaging-based interpretations rather than histologically confirmed biological identities.

**TABLE 4:** Imaging phenotype and prognostic association of the four MRI habitats.

Habitat	Putative biological interpretation	Dominant MRI pattern	Fraction, %, median IQR	Necrosis overlap, %	Edema overlap, %	HR for OS	95% CI	p value	FDR q value
Habitat 1	Enhancing-dominant habitat	T1ce high/FLAIR moderate	24.9 (15.9–35.5)	8.7 (3.2–16.6)	19.1 (10.3–29.9)	1.40	1.17–1.68	<0.001	0.003
Habitat 2	Necrotic-like habitat	T1ce low/T2 high/central low signal	18.5 (8.9–29.8)	47.1 (31.5–62.9)	12.5 (6.0–22.3)	1.74	1.38–2.19	<0.001	<0.001
Habitat 3	Peripheral edema-dominant habitat	FLAIR high/T1ce low/peripheral distribution	34.0 (22.6–46.7)	6.4 (2.0–13.9)	63.2 (48.3–77.0)	1.56	1.26–1.93	<0.001	0.002
Habitat 4	Mixed solid-tumor habitat	T1ce moderate/T2-FLAIR heterogeneous	21.5 (13.0–32.3)	14.9 (7.0–26.1)	24.8 (14.2–39.0)	0.82	0.67–1.00	0.046	0.049

Necrotic-like and edema-dominant habitats were the strongest adverse phenotypes. Habitat 2 had the highest HR for OS (HR = 1.74, 95% CI 1.38–2.19, FDR q < 0.001), and Habitat 3 was strongly associated with inferior survival as well (HR = 1.56, 95% CI 1.26–1.93, FDR q = 0.002). Habitat 4 showed a lower-risk pattern (HR = 0.82, 95% CI 0.67–1.00, FDR q = 0.049; Figures 3D,E and Table 4). These results suggest that survival risk was concentrated in specific spatial compartments rather than uniformly distributed across the tumor.

### Habitat-derived features and their associations with OS

**FIGURE 4:**
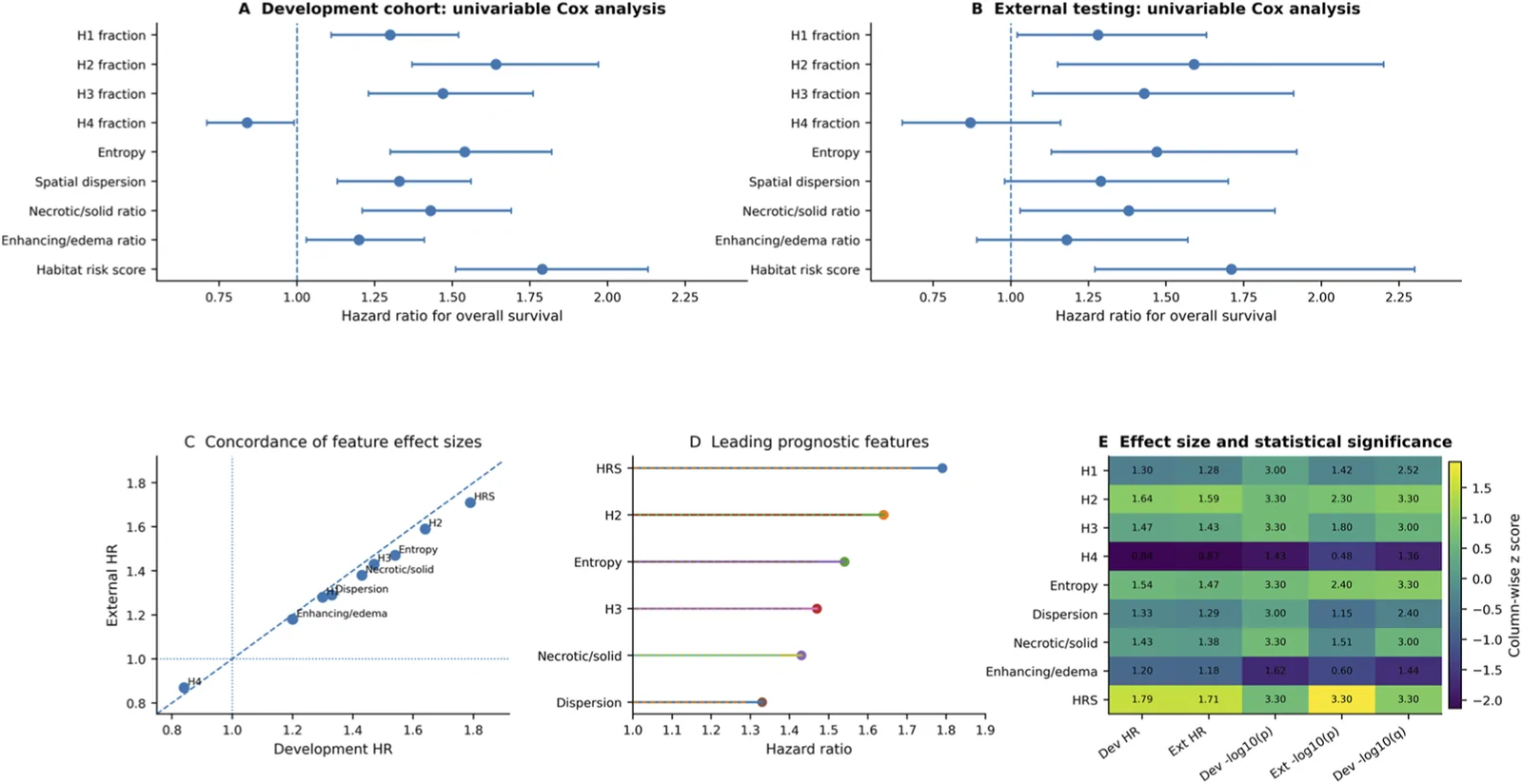
Prognostic associations of habitat-derived imaging features **(A)** Univariable Cox analyses in the development cohort **(B)** Corresponding analyses in the external testing cohort **(C)** Concordance of feature-specific hazard ratios across cohorts **(D)** Comparison of the leading prognostic features **(E)** Heat map summarizing effect sizes and statistical significance. All numerical values were aligned with Supplementary Table S1. HR, hazard ratio.

Continuous habitat-derived variables showed consistent prognostic associations. In the development cohort, Habitat 2 fraction was the strongest single habitat-fraction feature. Each 10% increase was associated with higher mortality risk (HR = 1.64, 95% CI 1.37–1.97, FDR q < 0.001). Habitat 3 fraction was also adverse (HR = 1.47, 95% CI 1.23–1.76, FDR q = 0.001), while Habitat 4 fraction showed a protective association (HR = 0.84, 95% CI 0.71–0.99, FDR q = 0.044; Figure 4A; Supplementary Table S1).

Higher-order heterogeneity descriptors also carried prognostic information. Habitat entropy was associated with poorer OS (HR = 1.54, 95% CI 1.30–1.82, FDR q < 0.001), as was the necrotic-to-solid habitat ratio (HR = 1.43, 95% CI 1.21–1.69, FDR q = 0.001). The integrated habitat risk score yielded the strongest overall effect size among the tested habitat-derived features in the development-cohort analysis (HR = 1.79, 95% CI 1.51–2.13, FDR q < 0.001; Figure 4A; Supplementary Table S1).

These associations generalized to the external testing cohort. Habitat 2 fraction remained associated with OS (HR = 1.59, 95% CI 1.15–2.20, p = 0.005), as did Habitat 3 fraction (HR = 1.43, 95% CI 1.07–1.91, p = 0.016), habitat entropy (HR = 1.47, 95% CI 1.13–1.92, p = 0.004), and the necrotic-to-solid habitat ratio (HR = 1.38, 95% CI 1.03–1.85, p = 0.031). The integrated habitat risk score showed the strongest and most consistent association in both cohorts (development HR = 1.79, 95% CI 1.51–2.13, FDR q < 0.001; external HR = 1.71, 95% CI 1.27–2.30, p < 0.001; Figures 4B–E; Supplementary Table S1).

### Habitat risk score: independent prognostic value and model performance

**FIGURE 5:**
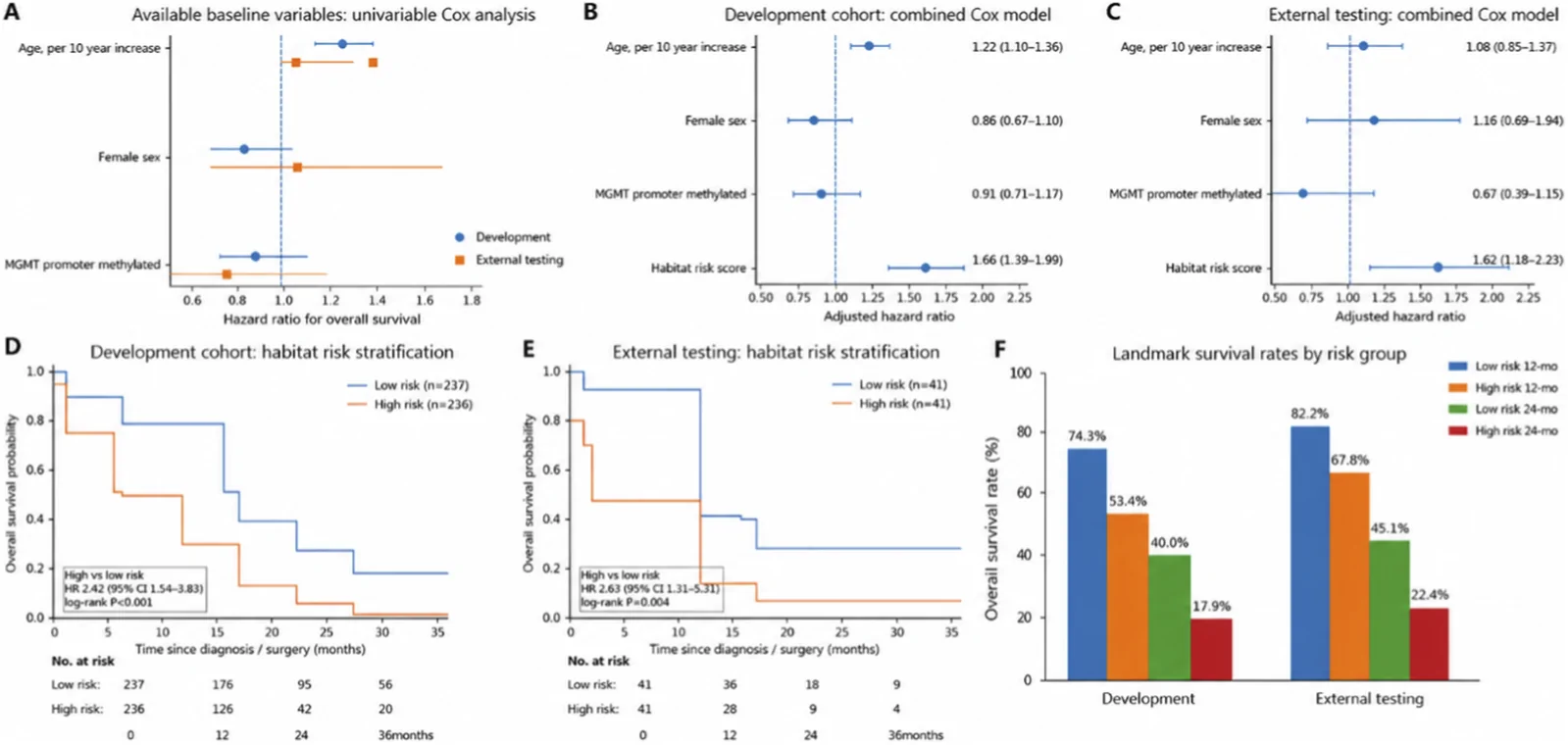
Independent prognostic value and survival stratification of the habitat risk score **(A)** Univariable Cox analyses of available baseline variables **(B,C)** Combined multivariable Cox models in the development and external testing cohorts **(D,E)** Kaplan-Meier curves for risk groups defined using the median risk score within each cohort **(F)** Twelve- and 24-month survival rates by risk group. HR, hazard ratio; OS, overall survival.

We then compared the habitat-derived signal with available baseline variables. In univariable Cox analysis, older age was associated with worse OS in the development cohort but not in the external testing cohort. Female sex was not significantly associated with OS in either cohort, while MGMT promoter methylation showed a directionally protective but non-significant association in both cohorts (Figure 5A; Supplementary Table S2).

The habitat risk score remained independently prognostic after adjustment for the consistently available baseline variables of age, sex, and MGMT promoter methylation. In the development cohort, the adjusted HR for the habitat risk score was 1.66 (95% CI 1.39–1.99, p < 0.001; Figure 5B; Supplementary Table S3). In the external testing cohort, the adjusted HR remained significant at 1.62 (95% CI 1.18–2.23, p = 0.003; Figure 5C; Supplementary Table S4). By contrast, available baseline variables showed weaker or less consistent associations across cohorts, indicating that habitat information captured prognostic variation not fully represented by age, sex, or MGMT status.

**FIGURE 6:**
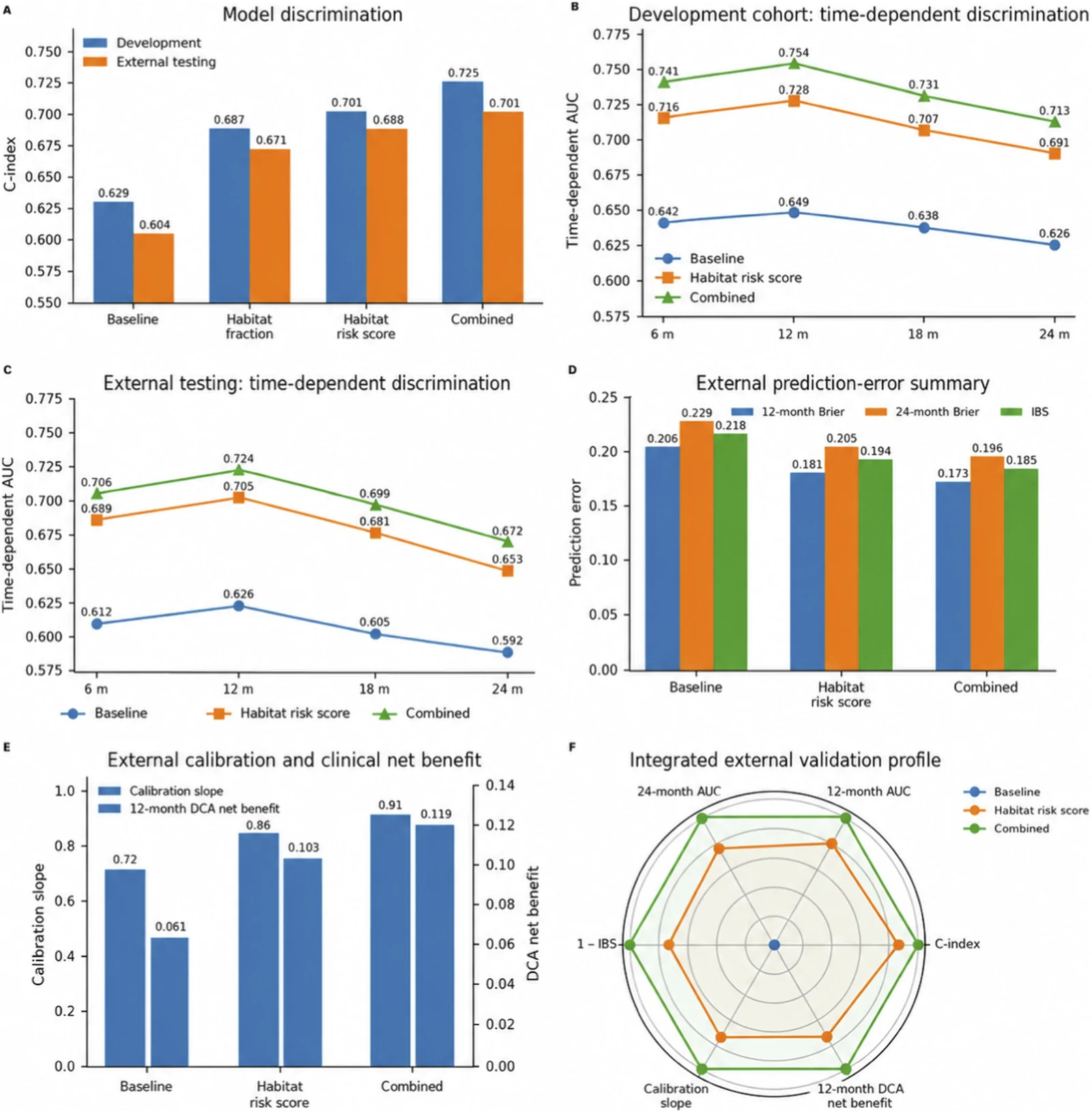
Model performance, calibration, and external validation **(A)** C-index comparison across model classes **(B,C)** Time-dependent AUCs in the development and external testing cohorts **(D)** External prediction-error metrics **(E)** External calibration slope (left axis) and 12-month decision-curve net benefit (right axis) **(F)** Integrated external validation profile; each metric was min-max normalized across the three models for visualization, and IBS was reversed as 1 - IBS so that higher values consistently indicate better performance. AUC, area under the receiver operating characteristic curve; IBS, integrated Brier score; DCA, decision curve analysis.

At the model level, the baseline model achieved an external C-index of 0.604 (95% CI, 0.526–0.682). The habitat risk score model improved the external C-index to 0.688 (95% CI, 0.617–0.759), while the combined model achieved the highest external C-index of 0.701 (95% CI, 0.632–0.770). Likelihood ratio tests supported the overall significance of the habitat and combined models, and the proportional hazards assumption was not evidently violated (Table 5). Compared with the baseline model, the combined model improved the external C-index by 0.097 (p = 0.031; Figure 6A; Supplementary Table S5).

**TABLE 5:** Overall model-level performance of multivariable Cox models.

Model	Variables included	Development C-index	External C-index	Development LR p	External LR p	PH assumption p	Main conclusion
Baseline model	Age + sex + MGMT promoter methylation status	0.629	0.604	0.005	0.169	0.418	Baseline variables have limited external stability
Habitat model	Habitat risk score	0.701	0.688	<0.001	<0.001	0.529	Habitat risk score independently predicts OS
Combined model	Age + sex + MGMT promoter methylation status + habitat risk score	0.725	0.701	<0.001	0.002	0.482	Habitat improves baseline model performance

Time-dependent AUC analysis revealed a similar pattern. In the development cohort, the baseline model achieved AUCs of 0.642, 0.649, 0.638, and 0.626 at 6, 12, 18, and 24 months, respectively. The combined model increased these values to 0.741, 0.754, 0.731, and 0.713, with an integrated AUC of 0.735 (Figure 6B; Supplementary Table S6). In the external testing cohort, the baseline, habitat risk score, and combined models achieved 12-month AUCs of 0.626 (95% CI, 0.530–0.722), 0.705 (95% CI, 0.616–0.794), and 0.724 (95% CI, 0.638–0.810), respectively. The corresponding 24-month AUCs were 0.592 (95% CI, 0.482–0.702), 0.653 (95% CI, 0.548–0.758), and 0.672 (95% CI, 0.570–0.774). The combined model achieved an integrated AUC of 0.701 and significantly outperformed the baseline model (p = 0.024; Figure 6C and Table 6; Supplementary Table S7).

**TABLE 6:** External testing performance of the baseline, habitat risk score, and combined models.

Model	n	Event n	C-index (95% CI)	12-month AUC (95% CI)	24-month AUC (95% CI)	IBS	Calibration slope	12-month DCA net benefit	p value vs. baseline
Baseline model	82	67	0.604 (0.526–0.682)	0.626 (0.530–0.722)	0.592 (0.482–0.702)	0.218	0.72	0.061	—
Habitat risk score model	82	67	0.688 (0.617–0.759)	0.705 (0.616–0.794)	0.653 (0.548–0.758)	0.194	0.86	0.103	0.038
Combined model	82	67	0.701 (0.632–0.770)	0.724 (0.638–0.810)	0.672 (0.570–0.774)	0.185	0.91	0.119	0.024

Prediction-error, calibration, and decision-curve analyses supported the added value of habitat information. In external testing, the integrated Brier score decreased from 0.218 for the baseline model to 0.194 for the habitat risk score model and 0.185 for the combined model. The 12-month Brier score decreased from 0.206 to 0.173 (Figure 6D; Supplementary Table S8). External calibration slope improved from 0.72 for baseline to 0.86 for the habitat model and 0.91 for the combined model, suggesting closer agreement between predicted and observed risk. The combined model also had the highest 12-month decision-curve net benefit in the external testing cohort, increasing from 0.061 to 0.119 (Figures 6E,F; Supplementary Table S8; Table 6).

The improvement was not limited to one isolated performance measure. The habitat risk score demonstrated enhancements in rank ordering of survival risk, time-specific prediction, prediction error, and calibration, while the combined model achieved the best overall external profile (Figure 6; Table 6). This broad pattern is particularly important in censored survival data because a higher AUC alone does not guarantee reliable risk estimation or clinical usefulness. The concurrent improvement in Brier score, calibration slope, and decision-curve net benefit suggests that habitat information strengthened both statistical predictive power and the potential for decision support.

### Risk stratification and sensitivity analyses

Habitat-derived risk stratification separated clinically distinct survival groups in both cohorts. In the development cohort, high-risk patients had a shorter median OS than low-risk patients (10.96 versus 22.41 months). The 12-month OS rate decreased from 74.3% in the low-risk group to 53.4% in the high-risk group, and the 24-month OS rate decreased from 40.0% to 17.9%. High-risk status was associated with substantially worse OS (HR = 2.42, 95% CI 1.93–3.03, log-rank p < 0.001), and the association remained significant after adjustment for baseline variables (adjusted HR = 2.16, p < 0.001; Figure 5D; Supplementary Table S9).

The same pattern was reproduced in the external testing cohort. Low-risk patients had a median OS of 28.46 months, whereas high-risk patients had a median OS of 12.91 months. The 12-month OS rate was 88.2% in low-risk patients and 67.8% in high-risk patients, while the 24-month OS rate was 45.1% in low-risk patients and 22.4% in high-risk patients. High-risk status was associated with worse OS (HR = 2.67, 95% CI 1.51–4.73, log-rank p < 0.001), and this remained significant after baseline adjustment (adjusted HR = 2.41, p = 0.002; Figures 5E,F; Supplementary Table S9).

External testing supported the added value of habitat-informed survival modeling. The baseline model showed modest discrimination, with C-index 0.604, 12-month AUC 0.626, 24-month AUC 0.592, and IBS 0.218. The habitat risk score model improved these metrics, achieving C-index 0.688, 12-month AUC 0.705, 24-month AUC 0.653, and IBS 0.194. The combined model achieved the best overall external profile, with C-index 0.701, 12-month AUC 0.724, 24-month AUC 0.672, and IBS 0.185. It also showed improved calibration and the highest decision-curve net benefit (Figure 6F; Table 6).

**FIGURE 7:**
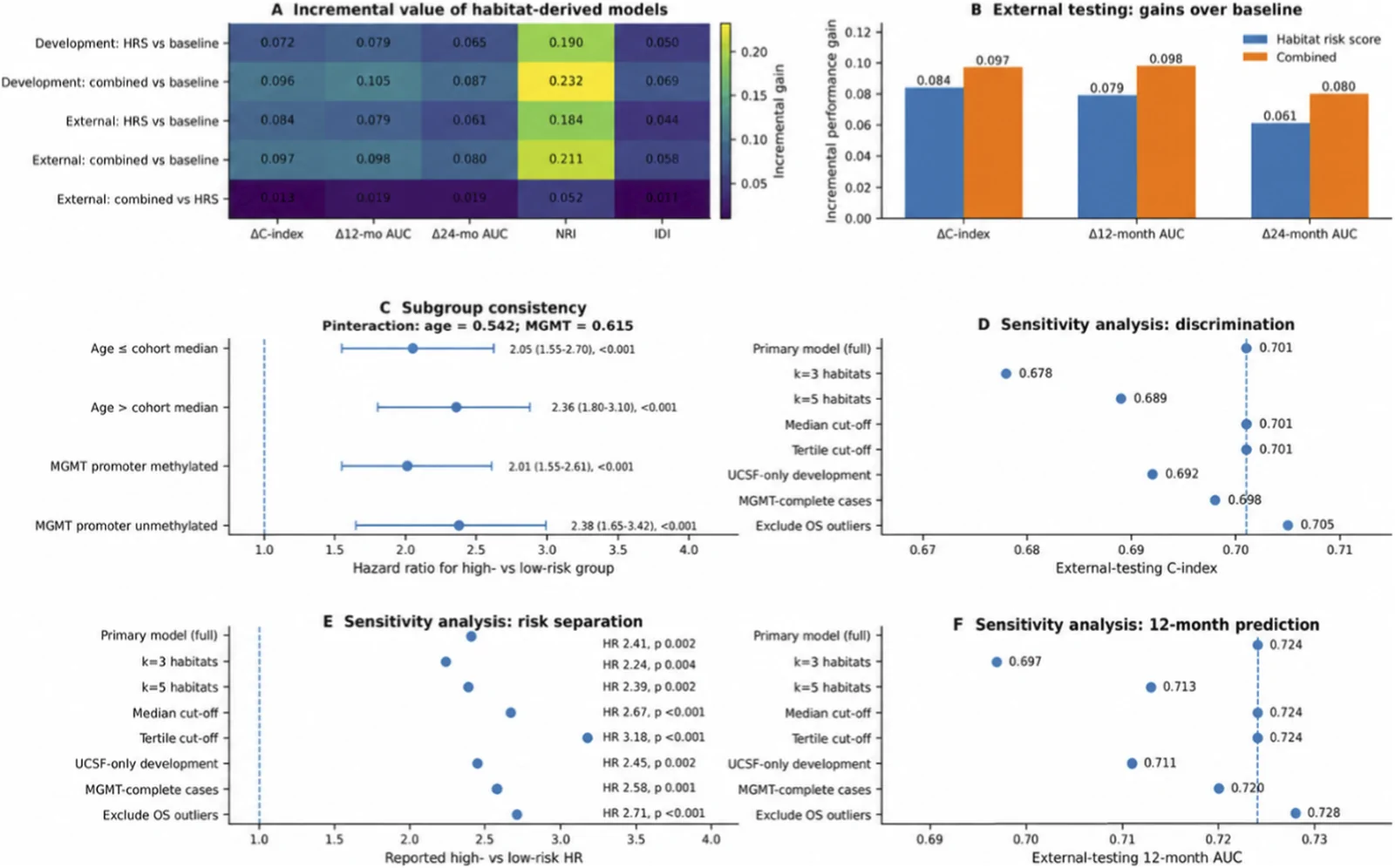
Incremental value, subgroup consistency, and sensitivity analyses **(A)** Incremental value of habitat-informed models **(B)** External performance gains over the baseline model **(C)** Age- and MGMT-defined subgroup analyses aligned with Supplementary Table S10 **(D–F)** Sensitivity analyses aligned with Supplementary Table S12. NRI, net reclassification improvement; IDI, integrated discrimination improvement; AUC, area under the receiver operating characteristic curve; HR, hazard ratio.

Subgroup analyses showed consistent adverse effects of high-risk status in age-defined and MGMT promoter methylation-defined subgroups, with no clear evidence of interaction. High-risk status remained associated with worse OS among patients aged at or below the cohort median and among those older than the cohort median. The habitat risk score also retained prognostic value in both MGMT-methylated and MGMT-unmethylated subgroups, indicating that its prognostic signal was not limited to a single age-defined or MGMT-defined subgroup (Figure 7C; Supplementary Table S10).

Incremental-value analyses confirmed measurable improvement over baseline models. In external testing, the combined model improved the C-index by 0.097, 12-month AUC by 0.098, and 24-month AUC by 0.080 compared with baseline. Significant gains were observed in net reclassification index (0.211, p = 0.035) and integrated discrimination index (0.058, p = 0.041; Figures 7A,B; Supplementary Table S11). These findings reinforce the central role of MRI-defined habitat heterogeneity in the proposed modeling framework.

Sensitivity analyses using k = 3 or k = 5, alternative risk cutoffs, public-database-only development, complete MGMT cases, and exclusion of OS outliers all preserved the direction and statistical significance of external survival separation (Figures 7D–F; Supplementary Table S12). These findings indicate that the prognostic value of MRI habitats was not dependent on a single modeling choice.

### Overall result synthesis

Overall, the results support a coherent prognostic model in which MRI-defined habitats capture clinically relevant spatial heterogeneity in GBM. The four-habitat solution was stable, interpretable, and survival-relevant. Necrotic-like and peripheral edema-dominant habitats were consistently associated with poorer OS. The integrated habitat risk score remained independently prognostic after adjustment for available baseline variables, improved model discrimination, reduced prediction error, improved calibration, and separated patients into clinically distinct risk groups in the development and external testing cohorts. These findings support MRI habitat analysis as a noninvasive approach for resolving prognostic spatial phenotypes in GBM.

## Discussion

This study demonstrates that MRI habitat analysis provides prognostic information by preserving the spatial organization of glioblastoma rather than averaging signal characteristics across the entire tumor. The four habitats represented distinct imaging phenotypes with different survival associations, suggesting that prognosis may be influenced by the relative distribution of viable tumor, necrosis, and peritumoral abnormality.

The enhancing-dominant habitat may reflect viable tumor tissue with blood–brain barrier disruption, increased vascularity, and active tumor burden (Barajas et al., 2012). The necrotic-like habitat showed the strongest adverse association with OS and may represent hypoxic, metabolically stressed, and treatment-resistant regions (Liu et al., 2017). The FLAIR-hyperintense peripheral habitat may contain a mixture of vasogenic edema and infiltrating tumor cells beyond the enhancing margin (He et al., 2024). In contrast, the relatively preserved solid-tumor habitat showed a lower-risk association, potentially reflecting a lower necrotic burden and less extensive peritumoral involvement. These interpretations remain imaging-based hypotheses because direct spatial histopathological or molecular validation was not available.

Clinically, the habitat risk score could complement routinely available prognostic variables for preoperative risk stratification. It may help identify patients requiring closer surveillance, support stratification in clinical trials, and guide future studies of habitat-directed biopsy or radiotherapy planning. However, the framework should not currently be used to determine treatment intensity, and prospective validation with treatment information and spatial pathological correlation is required before clinical implementation.

Another strength of this study is evaluation in an independent external cohort with a higher event proportion than the development cohort. This setting is clinically relevant because prognostic tools should maintain informative performance across different outcome distributions and institutional environments (Umehara et al., 2026). The preserved survival separation and improved calibration in the external testing cohort suggest that habitat-based risk assessment may be less dependent on the survival profile of a single development population than models based only on global imaging or baseline variables.

Several limitations should be acknowledged. First, several established prognostic factors for glioblastoma, including Karnofsky Performance Status, extent of resection, radiotherapy dose and schedule, temozolomide treatment details, TERT promoter mutation, and EGFR amplification, were not consistently available across all source cohorts and therefore could not be incorporated into the primary multivariable model. Consequently, the observed independent prognostic value of the habitat risk score should be interpreted as incremental value beyond the consistently available variables of age, sex, and MGMT promoter methylation, rather than beyond a complete clinical and molecular prognostic model. Residual confounding by treatment, functional status, surgical extent, and molecular characteristics cannot be excluded. Future prospective studies should collect these variables systematically and evaluate whether the habitat risk score retains prognostic value after adjustment for comprehensive clinical, treatment, surgical, and molecular factors. Second, although independent external testing was performed, the external cohort was moderate in size and came from a single institution. Third, MRI acquisition heterogeneity may still influence habitat delineation despite standardized preprocessing. Fourth, habitat labels were imaging-defined and biologically plausible but were not directly validated using spatial histopathology or molecular profiling (Buzdugan et al., 2026). Future work should combine longitudinal imaging, treatment-response data, and spatial molecular annotation to clarify whether habitat signatures can guide adaptive therapy or predict recurrence patterns (Pignotti et al., 2024).

## Conclusion

Preoperative MRI habitat analysis captures survival-relevant spatial heterogeneity in GBM. Necrotic-like and edema-dominant peripheral habitats emerged as key adverse compartments, and a composite habitat risk score improved prognostic modeling beyond available baseline variables. The externally validated performance and interpretable risk stratification support MRI habitat imaging as a promising noninvasive tool for GBM survival assessment, while prospective multicenter and biologically annotated validation remains necessary before clinical implementation.

## Data Availability

The raw data supporting the conclusions of this article will be made available by the authors, without undue reservation.
