# Supplementary material for: MRI-derived habitat heterogeneity for overall survival risk stratification in IDH-wildtype, CNS WHO grade 4 glioblastoma
Source: Front Cell Dev Biol. 2026 Sep 9;14:1917791. doi: 10.3389/fcell.2026.1917791 (PMC13597936; doi:10.3389/fcell.2026.1917791)
Supplement: Supplementary file 1 [file DataSheet1.docx]

Table S1. Univariable Cox analysis of habitat-derived features in the development and external testing cohorts.

| Feature | Feature type | Unit | Development HR | Development 95% CI | Development p value | Development FDR q value | External testing HR | External testing 95% CI | External testing p value |
| --- | --- | --- | --- | --- | --- | --- | --- | --- | --- |
| Habitat 1 fraction | Enhancing-dominant habitat | per 10% increase | 1.30 | 1.11-1.52 | 0.001 | 0.003 | 1.28 | 1.02-1.63 | 0.038 |
| Habitat 2 fraction | Necrotic-like habitat | per 10% increase | 1.64 | 1.37-1.97 | <0.001 | <0.001 | 1.59 | 1.15-2.20 | 0.005 |
| Habitat 3 fraction | Peripheral edema-dominant habitat | per 10% increase | 1.47 | 1.23-1.76 | <0.001 | 0.001 | 1.43 | 1.07-1.91 | 0.016 |
| Habitat 4 fraction | Mixed solid-tumor habitat | per 10% increase | 0.84 | 0.71-0.99 | 0.037 | 0.044 | 0.87 | 0.65-1.16 | 0.331 |
| Habitat entropy | Spatial heterogeneity | per 1 SD increase | 1.54 | 1.30-1.82 | <0.001 | <0.001 | 1.47 | 1.13-1.92 | 0.004 |
| Habitat spatial dispersion | Spatial distribution | per 1 SD increase | 1.33 | 1.13-1.56 | 0.001 | 0.004 | 1.29 | 0.98-1.70 | 0.071 |
| Necrotic-to-solid habitat ratio | Habitat ratio | per 1 SD increase | 1.43 | 1.21-1.69 | <0.001 | 0.001 | 1.38 | 1.03-1.85 | 0.031 |
| Enhancing-to-edema habitat ratio | Habitat ratio | per 1 SD increase | 1.20 | 1.03-1.41 | 0.024 | 0.036 | 1.18 | 0.89-1.57 | 0.254 |
| Habitat risk score | Integrated habitat score | per 1 SD increase | 1.79 | 1.51-2.13 | <0.001 | <0.001 | 1.71 | 1.27-2.30 | <0.001 |

Table S2. Univariable Cox analysis of available baseline variables.

| Variable | Definition | Available cases | Development HR | Development 95% CI | Development p value | External testing HR | External testing 95% CI | External testing p value |
| --- | --- | --- | --- | --- | --- | --- | --- | --- |
| Age | per 10-year increase | Development 473; External testing 82 | 1.27 | 1.14-1.41 | <0.001 | 1.05 | 0.85-1.32 | 0.637 |
| Female sex | female vs male | Development 473; External testing 82 | 0.82 | 0.64-1.04 | 0.096 | 1.07 | 0.66-1.74 | 0.775 |
| MGMT promoter methylated | MGMT promoter methylated vs unmethylated | Development 473; External testing 82 | 0.87 | 0.69-1.11 | 0.274 | 0.73 | 0.44-1.20 | 0.211 |

Table S3. Multivariable Cox regression in the development cohort.

| Variable | Baseline model HR | Baseline 95% CI | Baseline p value | Habitat model HR | Habitat 95% CI | Habitat p value | Combined model HR | Combined 95% CI | Combined p value |
| --- | --- | --- | --- | --- | --- | --- | --- | --- | --- |
| Age, per 10-year increase | 1.27 | 1.15-1.41 | <0.001 | - | - | - | 1.22 | 1.10-1.36 | <0.001 |
| Female sex | 0.84 | 0.66-1.07 | 0.165 | - | - | - | 0.86 | 0.67-1.10 | 0.232 |
| MGMT promoter methylated | 0.87 | 0.69-1.11 | 0.278 | - | - | - | 0.91 | 0.71-1.17 | 0.467 |
| Habitat risk score | - | - | - | 1.79 | 1.51-2.13 | <0.001 | 1.66 | 1.39-1.99 | <0.001 |

Table S4. Multivariable Cox regression in the external testing cohort.

| Variable | Baseline model HR | Baseline 95% CI | Baseline p value | Habitat model HR | Habitat 95% CI | Habitat p value | Combined model HR | Combined 95% CI | Combined p value |
| --- | --- | --- | --- | --- | --- | --- | --- | --- | --- |
| Age, per 10-year increase | 1.11 | 0.88-1.40 | 0.392 | - | - | - | 1.08 | 0.85-1.37 | 0.518 |
| Female sex | 1.23 | 0.74-2.06 | 0.427 | - | - | - | 1.16 | 0.69-1.94 | 0.574 |
| MGMT promoter methylated | 0.64 | 0.37-1.11 | 0.111 | - | - | - | 0.67 | 0.39-1.15 | 0.144 |
| Habitat risk score | - | - | - | 1.71 | 1.27-2.30 | <0.001 | 1.62 | 1.18-2.23 | 0.003 |

Table S5. Comparison of survival prediction performance across model classes.

| Model | Variables included | Development C-index | External testing C-index | Delta C-index vs baseline in External testing | p value vs baseline |
| --- | --- | --- | --- | --- | --- |
| Baseline model | Age + sex + MGMT promoter methylation status | 0.629 | 0.604 | Reference | - |
| Habitat fraction model | Habitat 1-4 fractions | 0.687 | 0.671 | +0.067 | 0.048 |
| Habitat risk score model | Habitat risk score | 0.701 | 0.688 | +0.084 | 0.026 |
| Combined model | Age + sex + MGMT promoter methylation status + habitat risk score | 0.725 | 0.701 | +0.097 | 0.031 |

Table S6. Time-dependent AUCs in the development cohort.

| Model | 6-month AUC | 12-month AUC | 18-month AUC | 24-month AUC | Integrated AUC | p value vs baseline |
| --- | --- | --- | --- | --- | --- | --- |
| Baseline model | 0.642 | 0.649 | 0.638 | 0.626 | 0.639 | - |
| Habitat risk score model | 0.716 | 0.728 | 0.707 | 0.691 | 0.710 | <0.001 |
| Combined model | 0.741 | 0.754 | 0.731 | 0.713 | 0.735 | <0.001 |

Table S7. Time-dependent AUCs in the external testing cohort.

| Model | 6-month AUC | 12-month AUC | 18-month AUC | 24-month AUC | Integrated AUC | p value vs baseline |
| --- | --- | --- | --- | --- | --- | --- |
| Baseline model | 0.612 | 0.626 | 0.605 | 0.592 | 0.609 | - |
| Habitat risk score model | 0.689 | 0.705 | 0.681 | 0.653 | 0.682 | 0.038 |
| Combined model | 0.706 | 0.724 | 0.699 | 0.672 | 0.701 | 0.024 |

Table S8. Prediction error and calibration performance of the survival models.

| Model | Development 12-month Brier | Development 24-month Brier | Development IBS | External testing 12-month Brier | External testing 24-month Brier | External testing IBS | External testing calibration slope | p value vs baseline |
| --- | --- | --- | --- | --- | --- | --- | --- | --- |
| Baseline model | 0.194 | 0.215 | 0.204 | 0.206 | 0.229 | 0.218 | 0.72 | - |
| Habitat risk score model | 0.166 | 0.190 | 0.179 | 0.181 | 0.205 | 0.194 | 0.86 | 0.041 |
| Combined model | 0.155 | 0.178 | 0.167 | 0.173 | 0.196 | 0.185 | 0.91 | 0.028 |

Table S9. Risk stratification performance of the habitat-derived risk score.

| Dataset | Risk group | n | Death n (%) | Median OS, months | 12-month OS rate | 24-month OS rate | HR | 95% CI | log-rank p | Adjusted HR | Adjusted p |
| --- | --- | --- | --- | --- | --- | --- | --- | --- | --- | --- | --- |
| Development | Low risk | 237 | 115 (48.5%) | 22.41 | 74.3% | 40.0% | Reference | - | - | Reference | - |
| Development | High risk | 236 | 171 (72.5%) | 10.96 | 53.4% | 17.9% | 2.42 | 1.93-3.03 | <0.001 | 2.16 | <0.001 |
| External testing | Low risk | 41 | 26 (63.4%) | 28.46 | 88.2% | 45.1% | Reference | - | - | Reference | - |
| External testing | High risk | 41 | 41 (100.0%) | 12.91 | 67.8% | 22.4% | 2.67 | 1.51-4.73 | <0.001 | 2.41 | 0.002 |

Table S10. Subgroup analysis of habitat risk stratification.

| Subgroup | n | Event n | Habitat C-index | HR high vs low risk | 95% CI | log-rank p | Interaction p |
| --- | --- | --- | --- | --- | --- | --- | --- |
| Age <= cohort median | 293 | 178 | 0.682 | 2.05 | 1.55-2.70 | <0.001 | 0.542 |
| Age > cohort median | 262 | 175 | 0.704 | 2.36 | 1.80-3.10 | <0.001 | 0.542 |
| MGMT promoter methylated | 338 | 213 | 0.678 | 2.01 | 1.55-2.61 | <0.001 | 0.615 |
| MGMT promoter unmethylated | 217 | 140 | 0.711 | 2.38 | 1.65-3.42 | <0.001 | 0.615 |

Table S11. Incremental value of habitat-based models over the baseline model.

| Comparison | Dataset | Delta C-index | p value | Delta 12-month AUC | p value | Delta 24-month AUC | p value | NRI | p value | IDI | p value |
| --- | --- | --- | --- | --- | --- | --- | --- | --- | --- | --- | --- |
| Habitat risk score model vs Baseline model | Development | +0.072 | <0.001 | +0.079 | <0.001 | +0.065 | <0.001 | 0.190 | <0.001 | 0.050 | <0.001 |
| Combined model vs Baseline model | Development | +0.096 | <0.001 | +0.105 | <0.001 | +0.087 | <0.001 | 0.232 | <0.001 | 0.069 | <0.001 |
| Habitat risk score model vs Baseline model | External testing | +0.084 | 0.026 | +0.079 | 0.038 | +0.061 | 0.049 | 0.184 | 0.047 | 0.044 | 0.052 |
| Combined model vs Baseline model | External testing | +0.097 | 0.031 | +0.098 | 0.024 | +0.080 | 0.046 | 0.211 | 0.035 | 0.058 | 0.041 |
| Combined model vs Habitat risk score model | External testing | +0.013 | 0.314 | +0.019 | 0.268 | +0.019 | 0.291 | 0.052 | 0.402 | 0.011 | 0.366 |

Table S12. Sensitivity analyses assessing robustness of the primary model.

| Sensitivity analysis | Development n | External-testing n | External-testing C-index | External-testing 12-month AUC | Reported high vs low risk HR | Reported p value | Main conclusion retained |
| --- | --- | --- | --- | --- | --- | --- | --- |
| Primary model: k=4 habitats + combined model | 473 | 82 | 0.701 | 0.724 | 2.41 | 0.002 | Yes |
| Using k=3 habitats | 473 | 82 | 0.678 | 0.697 | 2.24 | 0.004 | Yes |
| Using k=5 habitats | 473 | 82 | 0.689 | 0.713 | 2.39 | 0.002 | Yes |
| Using median cut-off for risk grouping | 473 | 82 | 0.701 | 0.724 | 2.67 | <0.001 | Yes |
| Using tertile cut-off, high vs low tertile | 473 | 82 | 0.701 | 0.724 | 3.18 | <0.001 | Yes |
| UCSF-PDGM-v5-only development | 354 | 82 | 0.692 | 0.711 | 2.45 | 0.002 | Yes |
| Cases with complete MGMT promoter methylation status only | 473 | 82 | 0.698 | 0.720 | 2.58 | 0.001 | Yes |
| Excluding extreme OS outliers | 463 | 80 | 0.705 | 0.728 | 2.71 | <0.001 | Yes |

**Table S13. Complete parameters of the habitat risk-score model.**

| Selected feature | Definition | Unit | Development-cohort mean | Development-cohort SD | LASSO-Cox coefficient |
| --- | --- | --- | --- | --- | --- |
| Habitat 2 fraction | Proportion of the tumor-bearing region classified as the necrotic-like habitat | % | 20.10 | 15.50 | 0.340 |
| Habitat 3 fraction | Proportion classified as the peripheral edema-dominant habitat | % | 34.60 | 17.90 | 0.260 |
| Habitat entropy | Shannon entropy of the habitat fractions | Dimensionless | 1.17 | 0.23 | 0.290 |
| Necrotic-to-solid habitat ratio | Habitat 2 fraction divided by Habitat 4 fraction | Dimensionless | 0.96 | 0.72 | 0.220 |
| Habitat 4 fraction | Proportion classified as the mixed solid-tumor habitat | % | 22.40 | 14.30 | −0.140 |

**Supplementary Figure S1.** **Representative pretreatment MRI examples illustrating voxel-wise intratumoral habitat generation.** Representative pretreatment contrast-enhanced MRI images from two patients are shown. (A, E) Original axial contrast-enhanced MRI images. (B, F) Tumor regions of interest (ROIs) outlined by red contours. (C, G) Corresponding voxel-wise habitat maps generated by unsupervised clustering of local radiomic features within the tumor ROI. (D, H) Overlay visualization of habitat maps on the original MRI images, demonstrating the spatial distribution and heterogeneity of the identified habitats. Four habitat subregions are displayed with different colors: Habitat 1 (purple), Habitat 2 (green), Habitat 3 (yellow), and Habitat 4 (blue).
